# Scholastic performance and functional connectivity of brain networks in children

**DOI:** 10.1371/journal.pone.0190073

**Published:** 2018-01-24

**Authors:** Laura Chaddock-Heyman, Timothy B. Weng, Caitlin Kienzler, Kirk I. Erickson, Michelle W. Voss, Eric S. Drollette, Lauren B. Raine, Shih-Chun Kao, Charles H. Hillman, Arthur F. Kramer

**Affiliations:** 1 Beckman Institute, University of Illinois at Urbana-Champaign, Urbana, Illinois, United States of America; 2 Department of Psychology, University of Iowa, Iowa City, Iowa, United States of America; 3 Department of Psychology, University of Pittsburgh, Pittsburgh, Pennsylvania, United States of America; 4 Department of Kinesiology, University of North Carolina at Greensboro, Greensboro, North Carolina, United States of America; 5 Northeastern University, Department of Psychology, Boston, Massachusetts, United States of America; 6 Northeastern University, Department of Health Sciences, Boston, Massachusetts, United States of America; 7 Northeastern University, Office of the Provost, Boston, Massachusetts, United States of America; University of Texas at Austin, UNITED STATES

## Abstract

One of the keys to understanding scholastic success is to determine the neural processes involved in school performance. The present study is the first to use a whole-brain connectivity approach to explore whether functional connectivity of resting state brain networks is associated with scholastic performance in seventy-four 7- to 9-year-old children. We demonstrate that children with higher scholastic performance across reading, math and language have more integrated and interconnected resting state networks, specifically the default mode network, salience network, and frontoparietal network. To add specificity, core regions of the dorsal attention and visual networks did not relate to scholastic performance. The results extend the cognitive role of brain networks in children as well as suggest the importance of network connectivity in scholastic success.

## Introduction

Scholastic performance during childhood can predict success in later years, including success in vocational, graduate, and professional studies [1, 2]. Performance on academic subjects such as mathematics and reading are monitored by standardized tests to determine educational program effectiveness, school funding, and academic placement. The quantification and prediction of scholastic success are important to maximize the potential of today’s youth.

One of the keys to understanding academic success is to determine the cognitive and neural processes that are involved in scholastic performance [3, 4]. Yet relatively little is known about the neurocognitive correlates of scholastic performance. Cognitive processes related to scholastic performance include general cognitive ability [5], intelligence [6], processing speed [7] and goal-directed executive control function [3, 8], particularly working memory and inhibition [8–10]. Recently, an event-related brain potential (ERP) that reflects neural activity involved in attention [11] and cortical gray matter thickness in the frontal cortex [12] have been found to relate to scholastic achievement.

The present study is the first to use a whole-brain connectivity approach to explore whether functional connectivity of resting state brain networks is associated with scholastic performance in 7- to 9-year-old children. The brain is organized into multiple, overlapping networks comprised of regionally separate but temporally connected brain regions. Functional connectivity is a promising approach for characterizing the nature of interactions among brain regions by considering brain areas not as independent, but rather as part of a larger context, as coordinated brain systems that support higher-level cognitive abilities [13]. Resting state functional connectivity methods measure the temporal coherence between spatially remote brain regions during wakeful rest [14]. For example, two regions with a positive correlation in signal over time are said to have high functional connectivity, and regions uncorrelated or negatively correlated are thought to be in separate, and sometimes competing, brain networks [15, 16]. Resting state connectivity has been shown to be a reliable method for measuring brain networks in children [17]. Each resting state network plays a role in behavior and cognition [18, 19] and, in fact, functional networks in resting state data closely resemble brain networks during task activation conditions [20]. The examination of the association between scholastic performance and functional connectivity patterns may provide insight into how neurodevelopmental processes support school achievement during childhood.

Normative maturation of functional brain networks helps provide a context to formulate predictions. In general, children show less integration of associated brain systems relative to adults [21–23], and the functional brain networks in children are more diffuse and undifferentiated than adult networks [23–25]. From childhood to adulthood, short-range cortical connections begin to develop into longer-range connections [23, 26–28]. Some of these previous studies are limited because they do not reveal brain-behavior relationships. In the present study, we test the relationship between resting state functional connectivity and school-based metrics in 7- to 9-year-old children.

We predicted that higher scholastic performance would be associated with greater functional connectivity of well-established brain networks that support higher-level cognitive functions [29]. Specifically, we focused on the default mode, salience, frontoparietal and dorsal attention networks, large-scale brain networks that require efficient communication between the frontal cortex and the rest of the brain [29]. As a control analysis, we also examined connectivity differences in the visual system, which is engaged in lower level visual processing. We predicted that functional connectivity differences between higher and lower academic performers were specific to higher-level brain networks.

## Methods

Children were recruited from schools in East-Central Illinois. Eligible participants were required to (1) be 7- to 9-years-old, (2) have an absence of school- related learning disabilities (i.e., individual education plan related to learning), adverse health conditions, physical incapacities, or neurological disorders, (3) qualify as prepubescent (Tanner pubertal timing score ≤2) [30], (4) report no use of medications that influence central nervous system function, (5) demonstrate right handedness (as measured by the Edinburgh Handedness Questionnaire) [31], (6) complete a mock MRI session successfully to screen for claustrophobia in an MRI machine, and (7) sign an informed assent approved by the Institutional Review Board of the University of Illinois at Urbana-Champaign. A legal guardian also provided written informed consent in accordance with the Institutional Review Board of the University of Illinois at Urbana-Champaign. The guardian was asked to provide information regarding participants’ socioeconomic status (SES), as determined by: (1) participation in free or reduced-price lunch program at school, (2) the highest level of education obtained by the mother and father, and (3) number of parents who worked full-time [32]. Participants also completed the Woodcock Johnson III paper and pencil task of General Intellectual Ability to assess intelligence quotient (IQ) [33].

The Institutional Review Board of the University of Illinois at Urbana-Champaign approved the present study. The MRI scans were obtained at the Biomedical Imaging Center of the Beckman Institute of the University of Illinois. Children completed the scholastic performance assessment on a separate day, and testing occurred in a quiet, sound attenuated room in a one-on-one setting. Children were compensated $15/ hour for MRI testing and $10/ hour for the neuropsychological testing. The present study included seventy-four children (44 girls and 30 boys, mean age = 8.7 years, age range 7.8–9.9 years, grades 2–5). See Table 1 for participant information.

**Table 1:** Participant information.

Variable	Mean (SD, range)
N	74 (44 females)
Age (years)	8.66 (0.577, 7.8–9.9)
Tanner^a^	1.38 (0.474, 1–3)
SES^b^ (median)	1.93 (0.800, 1–3)
IQ^c^ (standard score)	110.54 (12.76, 78–148)
Total Scholastic Performance^d^ (composite)	111.45 (14.448, 86–154)
Word Recognition^d^ (standard score)	112.17 (12.65, 80–139)
Reading Comprehension^d^ (standard score)	110.85 (13.99, 80–148)
Math Concepts and Applications^d^ (standard score)	108.36 (14.33, 80–150)
Math Computation^d^ (standard score)	109.14 (16.70, 79–160)
Written Expression^d^ (standard score)	105.72 (16.21, 40–134)
Listening Comprehension^d^ (standard score)	107.65 (13.55, 64–138)
Word Recognition^d^ (standard score)	112.18 (12.65, 80–139)

^a^Pubertal timing assessed using a modified Tanner Staging System [30].

^b^Socioeconomic Status. SES was determined by the creation of a trichotomous index based on three variables: child participation in a free or reduced-price meal program at school, the highest level of education obtained by the child’s mother and father, and the number of parents who worked full-time [32].

^c^Woodcock Johnson III task of General Intellectual Ability. Standard score based on a mean of 100 and a standard deviation of 15.

^d^Kaufman Test of Educational Achievement

### Scholastic performance

Scholastic performance was assessed with subtests from the Kaufman Test of Educational Achievement, Second Edition [34]. Standardized scores (Mean = 100, SD = 15) for reading (word recognition and reading comprehension) and mathematics (math concepts and applications and math computation), written language (written expression), and oral language (listening comprehension) were determined. A total scholastic achievement composite variable was calculated as word recognition + reading comprehension + math concepts and applications + math computation + written expression + listening comprehension. Kaufman Test of Educational Achievement, Second Edition subtests have very high internal consistencies, inter-rater reliabilities, and internal validity (r = 0.91–0.97).

Specifically, the word recognition subtest involved pronouncing words of gradually increasing difficulty. The reading comprehension subtest involved reading words and pointing to the corresponding picture, acting out the action of words, and answering questions about reading passages. The math concepts and application subtest consisted of basic math concepts such as comparing numbers and rounding numbers, as well as problems requiring algebra, calculus, and trigonometry (88 items). The math computation subtest was a paper and pencil test involving the addition, subtraction, multiplication, and division of whole numbers and fractions (72 items). The written expression subtest involved writing tasks in the context of an age-appropriate storybook format. The listening comprehension subtest involved listening to passages (played via CD) and then orally responding to questions asked by the examiner.

### Resting state functional brain networks

#### Imaging data acquisition

T2*-weighted resting state images were acquired with a fast echo-planar imaging (EPI) sequence with Blood-oxygen-level dependent (BOLD) contrast (TA [acquisition time] = 4 minutes 6 seconds, TR = 2s, TE = 25ms, flip angle = 90 degrees, 36 3.0 mm-thick slices acquired in ascending order, Grappa acceleration factor = 2, 92 × 92 matrix resolution, voxel size 2.6 x 2.6 x 3.0). Participants were asked to lay still with eyes closed during the resting state scan.

To assist with registration, high-resolution structural MR scans were acquired using a 3D MPRAGE (Magnetization Prepared Rapid Gradient Echo) T1-weighted sequence with 0.9 mm isotropic resolution (TR = 1900 ms; TE = 2.32 ms; TI = 900 ms [repetition/echo/inversion times]). All images were collected on a Siemens Magnetom Trio 3T whole-body MRI scanner with 12-channel receiver head coil (Siemens Medical Solutions; Erlangen, Germany).

#### Imaging data analysis

All imaging processing and analyses were carried out with a script library containing tools from FSL 5.0.4 (Functional Magnetic Resonance Imaging of the Brain’s Software Library, http://www.fmrib.ox.ac.uk/fsl), AFNI (http://afni.nimh.nih.gov/afni), FreeSurfer (http://surfer.nmr.mgh.harvard.edu), and MATLAB (The MathWorks, Natick, MA, USA) [35, 36]. Voxels containing non-brain tissue were stripped from the T1 structural images using FSL’s BET (Brain Extraction Technique) algorithm [37]. Each skull-stripped anatomical image was inspected.

For the resting state fMRI data, a six degree-of-freedom rigid-body head motion correction was applied to the fMRI data via AFNI’s *3dvolreg* function, which produced six parameters of head motion (root-mean-squares of translational and rotational movement: X, Y, Z, pitch, roll, and yaw directions) for subsequent regression of spurious variance. Non-brain tissue was removed using BET, and spatially smoothing using a 6.0 mm three-dimensional Gaussian kernel of full-width at half-maximum was applied. The pre-processed time series data were temporally filtered using AFNI’s *3dBandpass* to ensure that the fMRI data fell within the frequency band of .008 < *f* < 0.08 Hz. This helps reduce unwanted noise such as high frequency physiological signals (e.g., cardiac pulse) and low frequency scanner drift. The frequency band was chosen to best represent the spontaneous, low frequency fluctuation of the BOLD fMRI signal in the brain [38, 39].

Following temporal filtering, the mean time series was extracted from three sources of non-neuronal variance: white matter signal from a region in white matter structure, cerebrospinal fluid signal from a region in the lateral ventricle, and the global signal derived from a whole-brain mask. These nuisance signals were used as covariates to control for artifacts in the brain that may confound functional connectivity outcomes. With these three nuisance signals, the six head motion parameters obtained from the rigid body motion correction were bandpassed with the same temporal filter applied to the fMRI data and included as nuisance regressors [40]. Together, the nine bandpassed nuisance regressors (white matter, CSF, global, and motion parameters) were entered into a multiple regression as independent variables predicting the resting state fMRI data as a dependent variable using FSL’s FEAT tool.

Finally, using the residual time series data following the nuisance regression, volumes containing excessive head motion were scrubbed following a procedure described by Power and colleagues [41]. In general, motion-contaminated volumes above a frame-wise displacement threshold of 0.5 mm that coincided with BOLD signal changes were removed from subsequent functional connectivity analyses. On average, 1.4 ± 2.7 volumes were scrubbed, equivalent to 1.2 ± 2.3% of total volumes, which affected 18 of the 74 children included in the analysis. The whole-brain exploratory analysis based on the correlation of network seeds and all other voxels in the brain (described below) was performed on the cleaned time series data.

**Fig 1:**
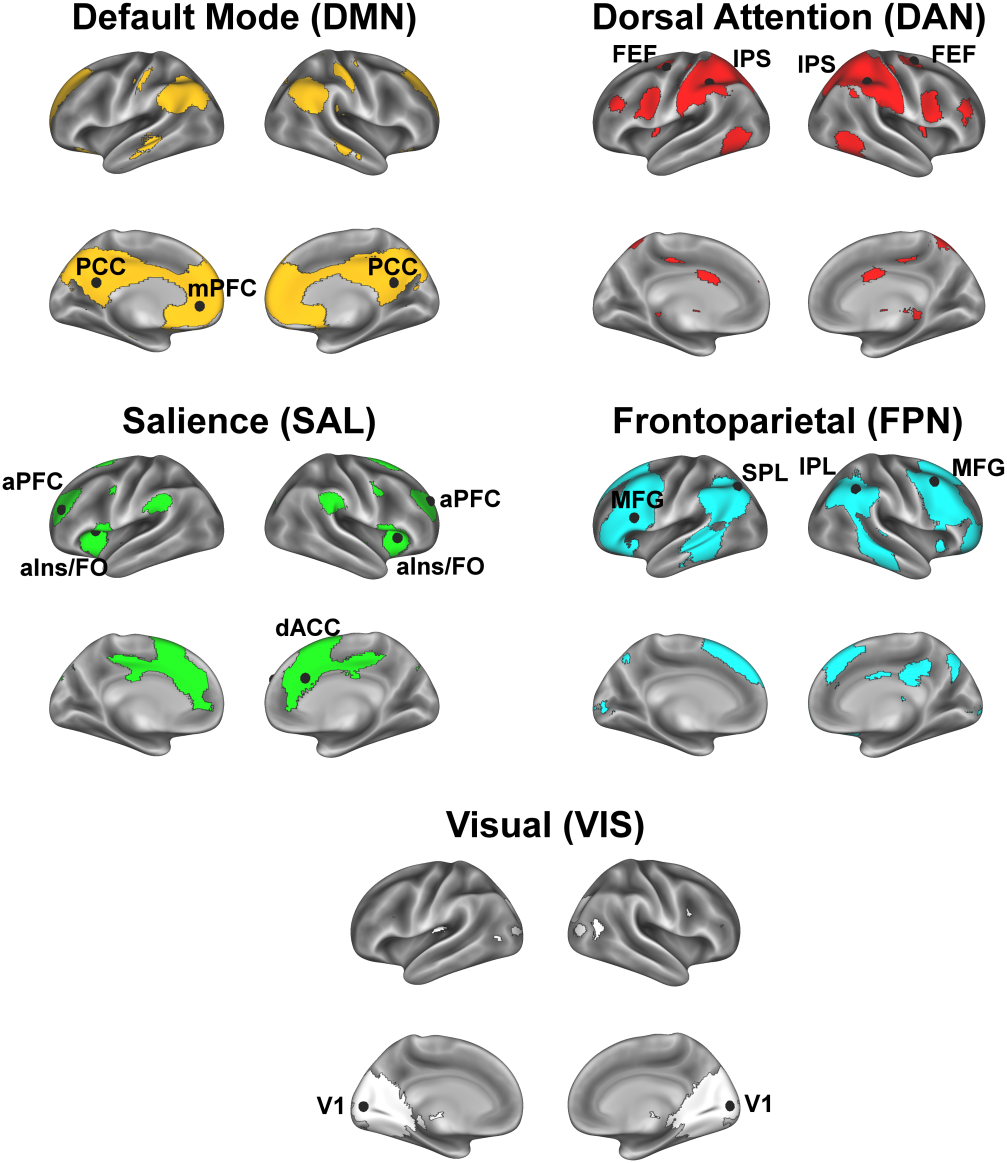
Illustration of group ICA that decomposed resting state fMRI data into independent spatiotemporal components, with core network ROIs. We identified ICs as cognitively relevant resting state networks and selected core ROIs that anchored each network (black spheres). Anterior and posterior DMN components were combined into one DMN network. DMN core: Posterior cingulate cortex (PCC), medial prefrontal cortex (mPFC); DAN core: Intraparietal sulcus (IPS), frontal eye field (FEF); SAL core: Anterior prefrontal cortex (aPFC), dorsal anterior cingulate cortex (dACC), anterior insula / frontal operculum (aIns/FO); FPN core: Middle frontal gyrus / dorsolateral prefrontal cortex (MFG), parietal lobule (inferior [IPL], superior [SPL]; VIS: Occipital pole (V1). The sample-specific template was derived from the group ICA, with each participant contributing a resting state scan to the ICA analysis (see Methods).

Our regions of interest (ROIs) for the targeted approach were derived from a group-level independent components analysis (ICA) that was applied to the pre-processed resting state fMRI data using FSL’s MELODIC with automatic dimensionality estimation. This data-driven analysis method decomposed the resting state fMRI data into 20 independent spatiotemporal components (IC) (Fig 1). Of the 20 ICs, we identified 6 ICs as cognitively relevant resting state networks that are established in the literature [17, 29, 42]: 1) anterior default mode network (DMN), 2) posterior DMN, 3) dorsal attention network (DAN), 4) left frontoparietal network (FPN), 5) right frontoparietal network (FPN), and 6) salience network (SAL). We also identified 1 IC resembling a sensory-related network: visual network (VIS). The remaining 14 ICs, which were excluded from our analyses, were deemed as nuisance artifacts or components not directly relevant to our hypotheses. See Fig 1 for the group ICA components included in the present study, and see S1 Fig for all group ICA components.

Guided by theory, we selected a core set of ROIs that strongly anchored each network and constructed 14-mm diameter spheres centered on each ROI’s peak coordinates in standard MNI (2 mm) space (Table 2) [43]. We parcellated each IC into distinct functional-anatomical clusters by performing a step-wise thresholding procedure beginning at Z > 2.33. For each IC, we increased the z-statistic until noncontiguous clusters emerged that reflected distinct regions known to comprise each network across studies in the existing literature. For example, for the DMN, this procedure parcellated the map into the dorsal medial prefrontal cortex, medial prefrontal cortex, left and right angular gyrus, left and right posterior cingulate cortex, left and right precuneus, and left and right superior frontal gyrus. Then, from these clusters, we located the peak statistical voxel in regions known to anchor each network (Table 2) [13, 36, 44, 45]. Finally, for each network, we merged these anchoring regions to form one network core mask (represented by black spheres in Fig 1) from which a mean timeseries across the regions was extracted for subsequent seed-based functional connectivity analyses.

**Table 2:** The core set of ROIs that anchored each network in order to characterize the associations between scholastic performance and functional connectivity between each network core and voxels throughout the brain.

Network	Description of anatomical region	MNI coordinates (x,y,z)
DMN	Posterior cingulate cortex	L: -6–54 20, R: 6–54 20
DMN	Medial prefrontal cortex	-2 50–4
DAN	Intraparietal sulcus	L: 40–38 44, R: 40–38 36
DAN	Frontal eye field	L: -36–10 52, R: 26–8 54
SAL	Anterior prefrontal cortex	L: -30 46 32, R: 30 50 32
SAL	Dorsal anterior cingulate cortex	0 26 32
SAL	Anterior insula / Frontal operculum	L: -38 16 4, R: 34 22 8
FPN	Middle frontal gyrus / dorsolateral prefrontal cortex	L: -50 14 32 R: 44 14 50
FPN	Inferior parietal lobule	R: 52–50 50
FPN	Superior parietal lobule	L: -38–66 50
VIS	Occipital pole	L: -6–92 8, R: 10–90 8

Individual EPIs were registered to high-resolution structural T1 space using the boundary-based registration (BBR) algorithm [46]. Registration of the EPIs from individual high-resolution structural space to standard MNI space was accomplished by FNIRT nonlinear registration with the default 10 mm warp resolution [47, 48]. The two resulting transformations were concatenated and then applied to the original functional image to create a functional image in standard MNI space; a reverse transform was used to register the seeds from standard MNI space to each participant’s native functional space.

Our primary aim was to characterize scholastic achievement-related differences in functional connectivity between each network core and voxels throughout the brain. For each participant, we conducted seed-based voxel-wise analyses using the network core masks as initiating seeds. First, the network masks were registered to native (functional) space using the transform described above. Then, we computed Pearson’s correlations between each seed’s mean time series and the time series at each voxel throughout the brain in native EPI space, resulting in a correlation map in which each voxel was designated a Pearson’s correlation coefficient representing the strength of correlation with the core seed. These whole-brain correlation maps were converted into z-score maps using a Fisher’s r-to-z transformation, resulting in subject-level Fisher’s z maps displaying voxels throughout the brain that were correlated with each ROI’s resting BOLD signal.

In preparation for group-level analyses, each subject-level voxelwise map was registered from native EPI space to the MNI152 template (2 mm resolution) by applying the (previously computed) EPI-to-MNI transform matrices. Once in standard space, the seed maps from individual subjects were concatenated to form a 4D image file (subject as the fourth dimension), and this 4D group image was input to group-level analyses using mixed-effects modeling as implemented in FSL’s *flameo* [49]. For each network, we tested whether individual differences in scholastic performance were related to individual differences in functional connectivity between the network’s core circuit and all other voxels in the brain. The mixed-effects model included demeaned scholastic performance scores as the variable of interest and covariates of no interest (SES, IQ). Multiple comparisons for the resulting group-level statistical maps were controlled by thresholding group contrast maps at Z>2.33, with cluster correction of p < 0.05 [50].

## Results

Scholastic performance was not associated with age, sex, or pubertal timing (all r <0.16, p>0.05). Given a significant association between total scholastic performance and SES (r = 0.258, p = 0.027), and total scholastic performance and IQ (r = 0.735, p<0.001), SES and IQ were included as covariates in all whole-brain regression models.

**Fig 2:**
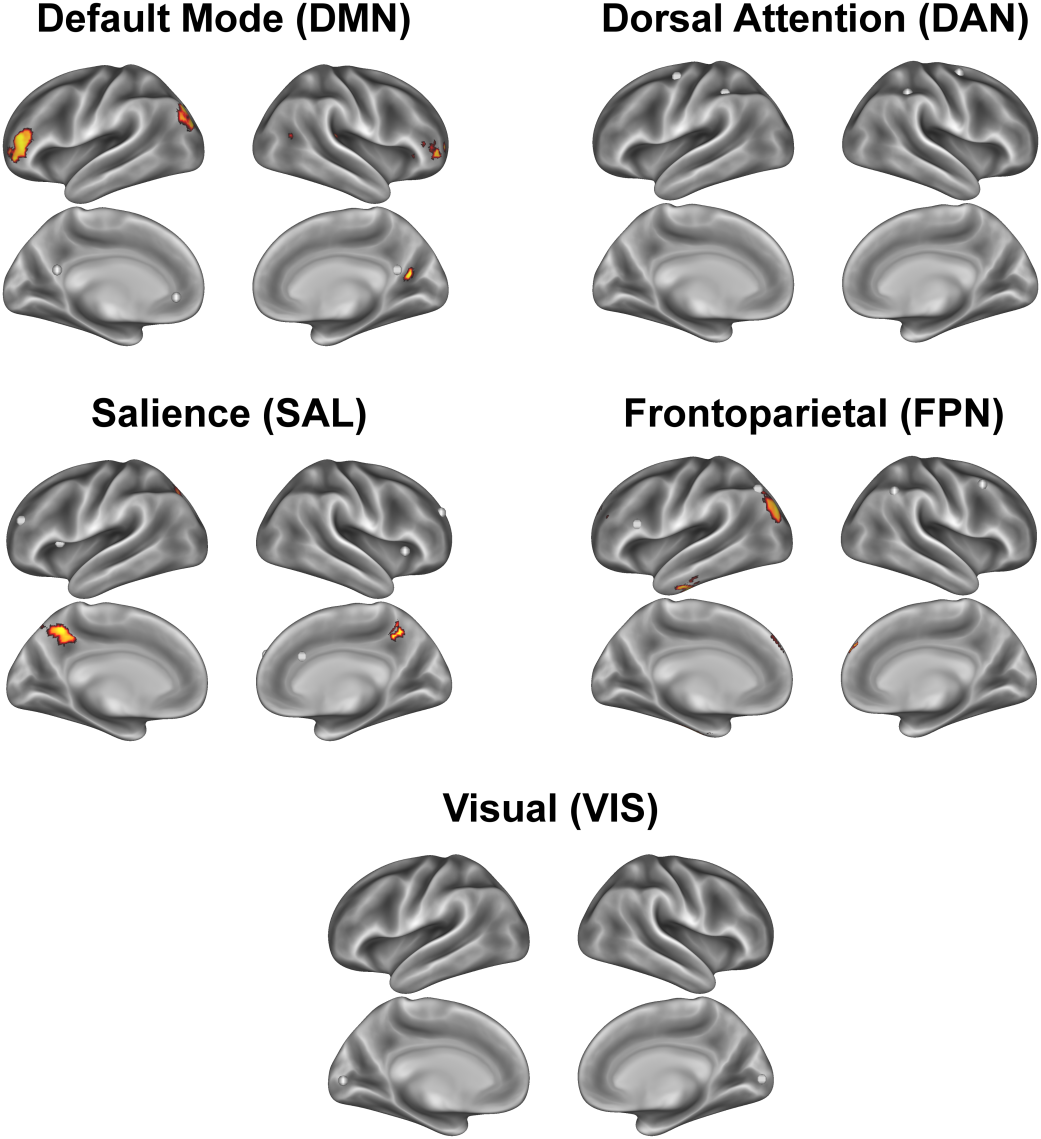
Total scholastic achievement-related differences in functional connectivity between each network core and voxels throughout the brain, via a mixed effects whole-brain model using FSL’s *flameo*. We illustrate positive associations (red/yellow clusters) between total scholastic performance and functional connectivity with core network regions (white spheres [Fig 1]). The red/yellow clusters represent the brain regions that children with higher scholastic achievement integrate into each functional brain network. Significant clusters survive threshold of Z > 2.3 and p < 0.05, corrected for multiple comparisons.

**Fig 3:**
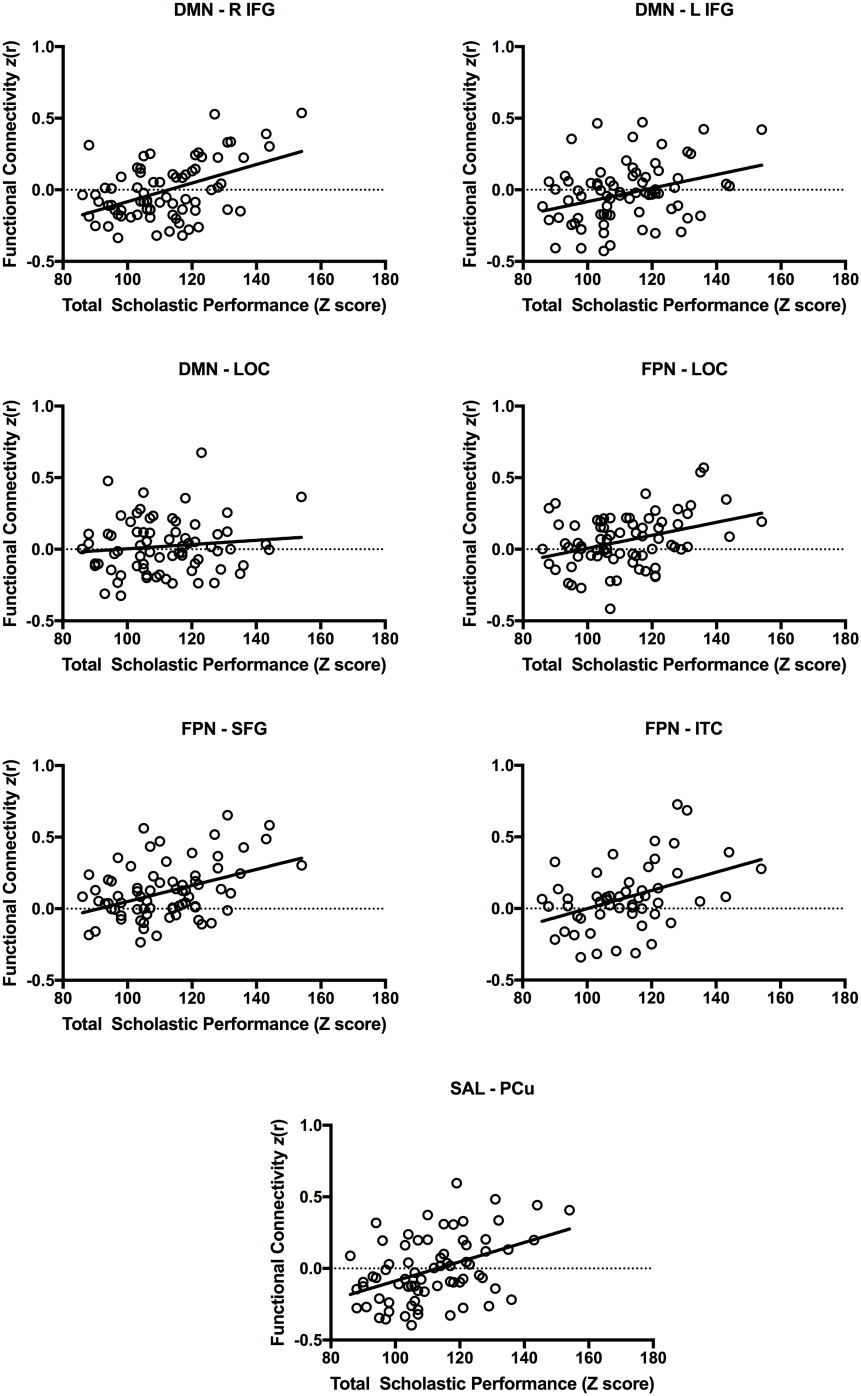
Representative scatterplots of the associations between total scholastic performance and functional connectivity. For each participant, we calculated functional connectivity between the core ROIs of the brain network with the significant brain region in the whole-brain *flameo* result.

For each network, we tested the positive association between total scholastic performance and functional connectivity between the network’s core circuit and all other voxels in the brain in a mixed effects whole-brain model. The whole-brain results demonstrated that higher scholastic performance was positively associated with greater functional connectivity between DMN core regions (posterior cingulate cortex, medial prefrontal cortex) and inferior frontal cortex and lateral occipital cortex (Fig 2, Table 3). Higher scholastic performance was positively associated with greater functional connectivity between SAL core regions (anterior prefrontal cortex, dorsal ACC, anterior insula/frontal operculum) and the precuneus (Fig 2, Table 3). Higher scholastic performance was positively associated with greater functional connectivity between FPN core regions (middle frontal gyrus/dorsolateral prefrontal cortex, superior and inferior parietal lobule) and superior frontal cortex, inferior temporal cortex, and lateral occipital cortex (Fig 2, Table 3). We did not observe significant associations between scholastic performance and functional connectivity in the DAN or VIS network. We included the peaks of all significant clusters (Table 3) as well as representative scatterplots of the associations between total scholastic performance and functional connectivity (Fig 3). See S2 Fig for associations between scholastic performance by specific academic subject (written language, mathematics, reading) and functional connectivity.

**Table 3:** Peak coordinates and Z-scores of significant clusters that correlate with functional connectivity within each network core. e.g., Children with higher scholastic performance show greater functional connectivity between the DMN core (posterior cingulate cortex and medial prefrontal cortex) and inferior frontal cortex and lateral occipital cortex.

Network	Description of anatomical region	MNI coordinates (x,y,z) (Z score)
DMN	Left inferior frontal cortex	-38 40 14 (Z = 3.75)
DMN	Right inferior frontal cortex	36 42 2 (Z = 3.51)
DMN	Lateral occipital cortex	-36–86 26 (Z = 3.48)
SAL	Precuneus	-6–52 48 (Z-3.79)
FPN	Superior frontal cortex	-18 58 36 (Z = 3.85)
FPN	Inferior temporal cortex	-42–10–36 (Z = 4.3)
FPN	Lateral occipital cortex	-32–78 32 (Z = 3.98)

## Discussion

The present study is the first to suggest an association between functional connectivity of resting state brain networks and scholastic performance in 7- to 9-year-old children. Children with higher total scholastic performance across reading, math and written and oral language have more integrated and interconnected resting state networks, specifically in the default mode network, salience network, and frontoparietal network. Specifically, children with higher scholastic performance show greater functional connectivity between the DMN core (posterior cingulate cortex and medial prefrontal cortex) and inferior frontal cortex and lateral occipital cortex, greater functional connectivity between the SAL core (anterior prefrontal cortex, dorsal anterior cingulate cortex, anterior insula/frontal operculum) and precuneus, and greater functional connectivity between the FPN core (middle frontal gyrus/dorsolateral prefrontal cortex, superior and inferior parietal lobule) and superior frontal cortex, inferior temporal cortex, and lateral occipital cortex. These associations were circuit specific such that core regions of the DAN and VIS networks did not relate to scholastic performance.

The results extend the cognitive role of brain networks in children by demonstrating an association between resting state functional connectivity and scholastic achievement. The DMN, SAL, and FPN are known to play a role in executive function (e.g., attention, inhibition) [51–55]. Executive function is linked to academic success [3,8]. In particular, successful school performance and problem solving are said to involve working memory, the ability to hold relevant information in mind for efficient and effective comprehension [8, 9, 56], as well as inhibition, the ability to ignore irrelevant information and inhibit inappropriate responses [57]. Indeed, networks that relate to school performance are implicated in these executive processes. For example, DMN activity is known to facilitate cognitive control, which includes working memory, inhibition, and cognitive flexibility [58, 59]. We are the first to extend the role of the DMN to include scholastic performance. Further, developmental changes in the DMN are not uniform across nodes [60]. Convergent evidence from structural and functional connectivity analyses suggest that connectivity between the posterior cingulate cortex and medial prefrontal cortex along the cingulum bundle is the most immature connection of the DMN in children [60]. The results of the present study suggest that children with higher scholastic performance have a more functionally connected posterior cingulate-frontal circuit. It is possible that children with higher scholastic performance show a shift toward a more adult state of functional organization [60].

We also report that scholastic performance relates to functional connectivity of a team of executive control networks (SAL and FPN). The SAL network is known to be important for sustained task-set maintenance, error feedback for tuning top-down control, and maintaining associations between actions and outcomes [61, 62]. In other words, this network detects stimuli that are consistent with behavioral goals (i.e., salience) and then facilitates the involvement of attentional and working memory resources via recruitment of other large-scale brain networks [51, 55]. The FPN supports goal-directed cognition by directing attention to salient stimuli and maintaining relevant data in mind until actions are selected [52]. Using this framework, it is possible that higher scholastic performers, with increased functional connectivity in the SAL network, may be better able to detect behaviorally relevant information. The frontoparietal executive network of higher achievers may also be more qualified to direct attention and memory processes, and to exert top-down control to execute an appropriate behavioral response, which results in higher scholastic performance.

Our results suggest a unique association between academic skills and functional connectivity of specific regions, rather than global effects across all brain networks. We do not demonstrate associations between achievement and connectivity within the DAN or VIS networks. The DAN, centered on the dorsal posterior parietal and frontal cortex, is known to play a role in the control of visual and spatial attention [63, 64]. The network is involved in both preparatory elements of cognitive selection of sensory information, as well as response and action selection, such that the network sends top-down signals regarding the processing of relevant stimulus features while taking into account goals and existing information. Our study suggests that functional connectivity with core areas of this network, particularly the intraparietal sulcus and frontal eye field, are not significantly related to scholastic performance in 7- to 9-year-old children. As predicted, scholastic performance did not relate to connectivity within the visual network (occipital pole).

Until recently, links between school achievement and executive control functions have mostly been explored via task performance or in-class observation [65, 66]. It is important to understand the role of brain health. Here, we build upon previous work using neuroelectric indices of attention and inhibition [11] and brain structure of the frontal cortex [12] by examining the role of functional connectivity of brain networks in scholastic success. Functional brain networks may be another pathway involved in school performance during development. Future research should investigate the specific associations among academic skills, executive control, and brain function and structure, as well as examine these associations across time. For example, one study demonstrated that functional connectivity and brain structure in frontal, parietal and temporal cortex at age 8 predicted gains in numerical abilities across the next 6 years [67]. Remarkably, behavioral measures of academic performance did not predict these longitudinal gains [67]. Our work provides a platform for additional researchers to examine how brain function and brain structure [68] relate to academic skills across a larger age range of children (given that our age range was limited to 7- to 9-year-old children). Furthermore, as our voxel-wise analyses were dependent on, and limited to, regions derived from a data-driven analysis, which allowed us to test targeted questions about the associations between scholastic performance and cognitively- and clinically-relevant functional networks [35, 36], future work may test whether sub-systems within or across brain networks are more involved in academics than the larger networks. In addition, as we applied a broad network level approach, graph theory could provide insight into specific connections between regions that relate to scholastic performance.

In conclusion, there are important implications of exploring the associations between brain health and scholastic performance, as standardized test performance can determine funding and effectiveness of educational programs as well as forecast a student’s future scholastic success [1, 2]. We show that functional connectivity of resting state brain networks has important scholastic implications in 7- to 9-year-old children, thereby extending our understanding of how neurodevelopmental processes support school success during childhood.

## Supporting information

S1 Fig Illustration of all components in group ICA. We labeled the ICs as: (1) Sensory-Motor Network, (3) Primary Visual Network, (4) Thalamus, (5) Secondary Visual, (6) DAN*, (7) Auditory, (9) Anterior Prefrontal, (10) Corpus Callosum, (11) SAL*, (12) Lateral occipital, (13) Anterior DMN*, (14) Cerebellum/Brainstem, (15) Posterior DMN*, (16) Cerebellum/Brainstem, (17) Right FPN*, (18) Left FPN*.
(HTML)

S2 Fig Individual scholastic achievement-related differences in functional connectivity between each network core and voxels throughout the brain, via a mixed effects whole-brain model using FSL’s *flameo*. Reading Composite = Word recognition + Reading comprehension. Mathematics Composite = Math Concepts, Applications, and Computation. Written Language Composite = Written Expression + Spelling. We illustrate positive associations (red/yellow clusters) between scholastic performance (by subject) and functional connectivity with core network regions (white spheres [Fig 1]). IQ was included as a covariate for all achievement scores, and SES for only mathematics. The red/yellow clusters represent the brain regions that children with higher scholastic achievement (written language, reading, mathematics) integrate into each functional brain network. Significant clusters survive threshold of Z > 2.3 and p < 0.05, corrected for multiple comparisons.
(PNG)
