# Supplementary material for: Scholastic performance and functional connectivity of brain networks in children
Source: PLoS One. 2018 Jan 24;13(1):e0190073. doi: 10.1371/journal.pone.0190073 (PMC5783351; doi:10.1371/journal.pone.0190073)
Supplement: S1 Fig — We labeled the ICs as: (1) Sensory-Motor Network, (3) Primary Visual Network, (4) Thalamus, (5) Secondary Visual, (6) DAN*, (7) Auditory, (9) Anterior Prefrontal, (10) Corpus Callosum, (11) SAL*, (12) Lateral occipital, (13) Anterior DMN*, (14) Cerebellum/Brainstem, (15) Posterior DMN*, (16) Cerebellum/Brainstem, (17) Right FPN*, (18) Left FPN*. (HTML) [file pone.0190073.s001.html]

Attachment not opening? Click this link: S1\_Fig.tif
